# Radiological features of intrathoracic chronic expanding hematoma: A case report

**DOI:** 10.1016/j.ijscr.2023.108767

**Published:** 2023-08-29

**Authors:** Mayu Inomata, Shoei Kuroki, Hiroshi Nakada, Fumiya Kawano, Ryo Maeda

**Affiliations:** a Department of Thoracic and Breast Surgery, Faculty of Medicine, University of Miyazaki, Miyazaki, Japan; b Department of Radiology, Faculty of Medicine, University of Miyazaki, Miyazaki, Japan

**Keywords:** Case report, Chronic expanding hematoma, Intrathoracic, Surgery

## Abstract

**Introduction and importance:**

We present a relatively rare case of intrathoracic chronic expanding hematoma (CEH) after thoracic surgery for lung cancer. CEH is often difficult to distinguish from malignant tumors because of its large size and slow progressive enlargement. In this report, we describe the radiological features of CEH in detail.

**Case presentation:**

A 67-year-old man who underwent a left upper lobectomy for lung cancer at 46 years of age presented with hemosputum. Computed tomography revealed a large mass with central low attenuation. Calcification was detected in peripheral lesions of the mass. T2-weighted magnetic resonance imaging (MRI) revealed a mass with mixed low and high signal intensities. Based on the clinical course, the patient was diagnosed with an intrathoracic CEH. A left posterolateral thoracotomy was performed with the patient in the lateral position, and a mass encased in a tough capsule was resected. The postoperative histopathological findings were consistent with CEH.

**Clinical discussion:**

CT of intrathoracic CEH shows a lesion with heterogeneous content, a thick wall, and calcifications. However, differentiation from malignant tumors is difficult using CT alone. MRI is a good diagnostic modality for CEH and often shows a mixture of low- and high-intensity areas on T2-weighted images. In addition, the patient's medical history is important because most cases of CEH have a history of trauma or surgery.

**Conclusion:**

To diagnose intrathoracic CEH, it is essential to consider the patient's clinical course and MRI findings.

## Highlights

• We present a case of intrathoracic chronic expanding hematoma (CEH) after thoracic surgery and describe its radiological features in detail.
• To diagnose intrathoracic CEH, it is essential to consider the patient’s clinical course and magnetic resonance imaging findings.

## 1 Introduction

Hematomas usually reabsorb and decrease in size slowly over time. In rare cases, a hematoma may slowly increase in size after an initial hemorrhagic event, referred to as a chronic expanding hematoma (CEH) [1]. CEH can develop in any body region [[2], [3], [4]] and is often difficult to distinguish from malignant tumors because of its large size and slow progressive enlargement [5]. Herein, we present a relatively rare case of intrathoracic CEH after thoracic surgery for lung cancer more than 20 years prior and describe its radiological features in detail. This surgical case has been reported in accordance with the SCARE 2020 guidelines [6].

## 2 Case presentation

**Fig. 1:**
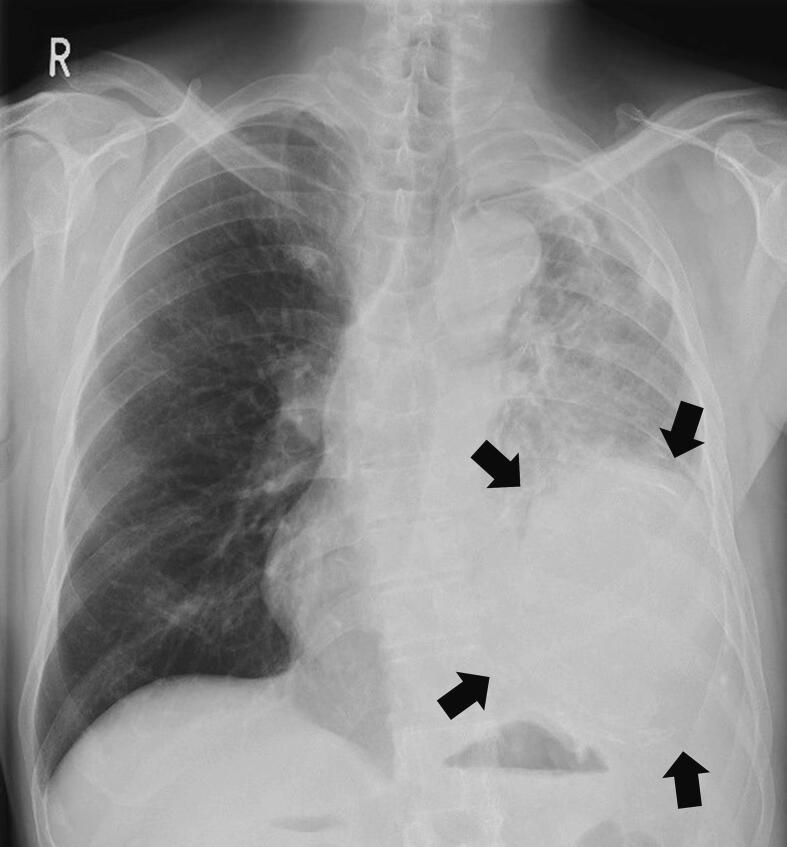
Chest roentgenogram depicting a huge mass shadow (black arrows) in the left lower lung field.

**Fig. 2:**
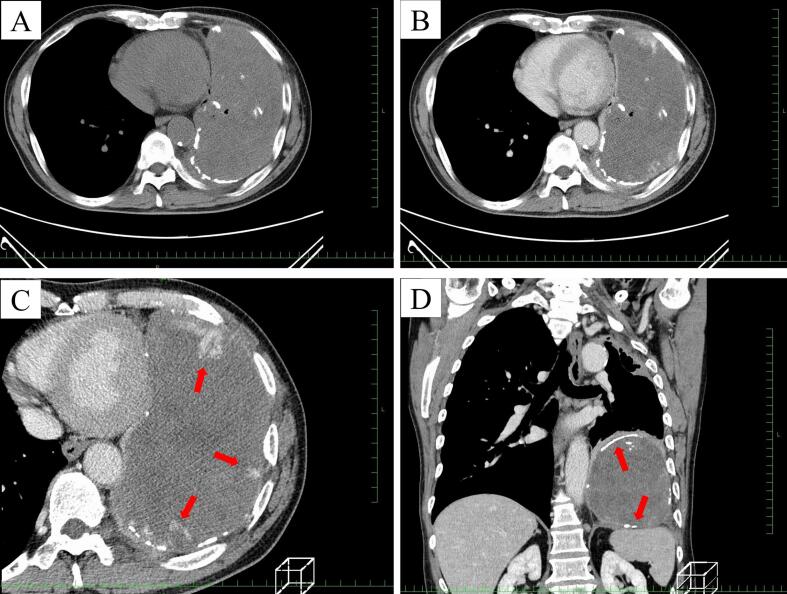
(A) Computed tomographic (CT) scan of the chest showing a large mass with central low attenuation measuring 16 × 12 × 8 cm. (B) Contrast-enhanced CT scan showing non-homogeneous enhancement. (C) Newly formed vessels (red arrows) inside the lesion on the thoracic side. (D) Calcification (red arrows) detected at peripheral lesions of the mass. (For interpretation of the references to colour in this figure legend, the reader is referred to the web version of this article.)

**Fig. 3:**
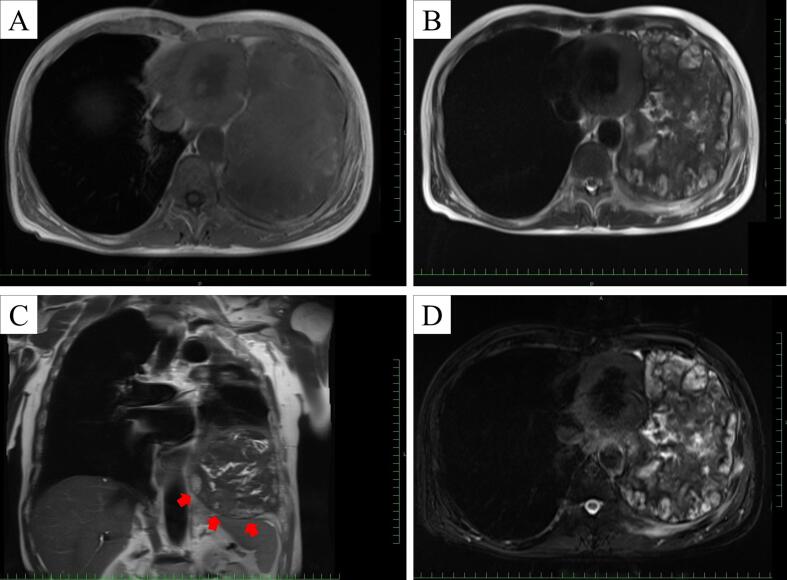
(A) T1-weighted magnetic resonance imaging shows a mass with predominantly low signal intensity. (B) Mosaic of various signal intensities on T2-weighted images. (C) Thick pseudocapsule (red arrows) with low intensity on T2-weighted images. (D) High signal intensity areas on T2-weighted images were not suppressed on fat-suppressed T2-weighted images. (For interpretation of the references to colour in this figure legend, the reader is referred to the web version of this article.)

**Fig. 4:**
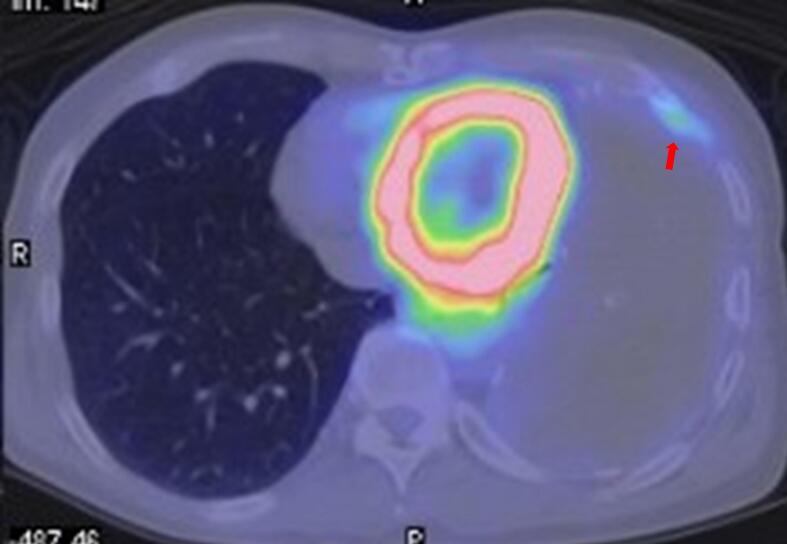
2-[Fluorine-18]fluoro-2-deoxy-d-glucoseuptake is only observed in the peripheral rim of the space-occupying mass (red arrow), most of which shows no uptake. (For interpretation of the references to colour in this figure legend, the reader is referred to the web version of this article.)

A 67-year-old man presented with a three-month history of hemosputum. Chest roentgenography revealed a large mass shadow in the left lower lung field (Fig. 1). The patient had undergone left upper lobectomy for lung cancer at 46 years of age. Computed tomography (CT) revealed a large mass measuring 16 × 12 × 8 cm with low central attenuation (Fig. 2A). Contrast-enhanced CT showed nonhomogeneous enhancement (Fig. 2B) and newly formed vessels inside the lesion (Fig. 2C). Calcification was detected at the periphery of the mass (Fig. 2D). T1-weighted magnetic resonance imaging (MRI) revealed a mass with predominantly low signal intensity (Fig. 3A), whereas T2-weighted MRI revealed a mass with mixed low and high signal intensities (Fig. 3B). A thick pseudocapsule characterized by low intensity on T1- and T2-weighted images was also observed (Fig. 3C). Areas with high signal intensity on T2-weighted images were not suppressed on fat-suppressed T2-weighted MRI (Fig. 3D). The heterogeneous signal intensity inside the lesion was assumed to represent differently aged hemorrhages within the hematoma. Positron emission tomography (PET) with 2-[18 F] fluoro-2-deoxy-d-glucose (FDG) images revealed uptake only in the peripheral rim of the mass (Fig. 4). The maximum standardized uptake value (SUV) of the lesion was 3.9. No other signs of abnormal uptake suggestive of malignancy were observed.

**Fig. 5:**
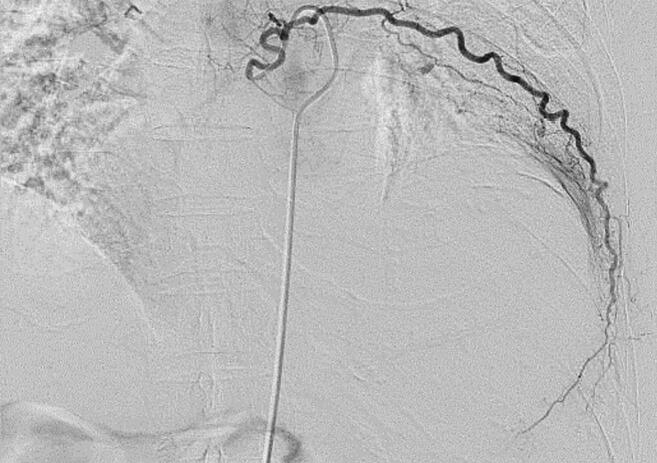
Arteriography shows the development of abnormal blood vessels distributed to the membrane of the mass from the 8th intercostal artery.

**Fig. 6:**
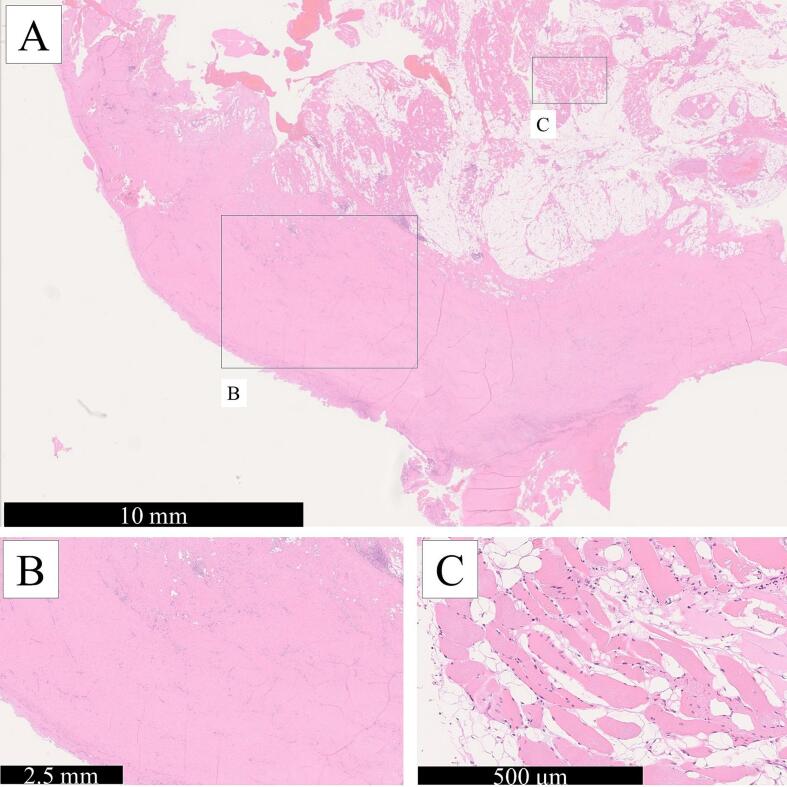
(A) Histological analysis reveals an old hematoma surrounded by fibrous tissue, with dilated microvessels and blood stasis within the hematoma (hematoxylin and eosin stain). (B) A thick fibrous capsule is noted (hematoxylin and eosin stain). (C) Observation of the inner region of the hematoma. No malignant findings are noted (hematoxylin and eosin stain).

Based on the clinical course, the patient was diagnosed with an intrathoracic CEH. Embolization of the vessels supplying the lesion was performed one day before the operation. Arteriography showed the development of abnormal blood vessels distributed to the membrane of the mass from the 8th intercostal artery (Fig. 5), which was embolized with Gelfoam particles. We performed a left posterolateral thoracotomy through the lateral position. The tumor was completely adherent to the chest wall, pericardium, left diaphragm, left lower lobe, and descending aorta, and had a tough capsule. The mass and tough capsule were resected as completely as possible. However, the capsule surrounding the descending aorta could not be completely removed because of its tight adherence. Total intraoperative blood loss was approximately 2000 mL. Macroscopic observation demonstrated a large, encapsulated mass with necrotic tissue and hemorrhagic material with calcification. The postoperative histopathological findings were consistent with CEH (Fig. 6). The patient was discharged from the hospital on the 13th postoperative day. No recurrence was observed nine months after the operation.

## 3 Discussion

CEH is an uncommon clinical entity initially described by Reid et al. [1] as an organized collection of blood that increases in size more than one month after an initial hemorrhagic event, such as trauma or surgery, not only in the thorax but anywhere in the body. Many cases of intrathoracic CEH have been reported as long-term tuberculosis-associated complications after artificial pneumothorax or tuberculous pleurisy [[7], [8], [9]]. In the present case, CEH developed more than 20 years after thoracotomy for lung cancer. In 6 reported cases of CEH in the thorax by Muramatsu et al., the latency period, defined as the time from the onset of the previous disorder until the manifestation of CEH symptoms, was approximately 40 years (range: 12–55 years) [10]. Artificial Intelligence in follow up may be helpful in early management and diagnosis for CEH [11].

The pathogenetic mechanism of CEH is not fully understood, but it has been suggested that surgery and trauma induce microhematoma formation, resulting in the release of inflammation-inducing substances and degradation products released from various blood components [12]. This promotes neovascularization with repeated hemorrhages and capsule formation. In the thorax, vulnerable blood vessels are likely to collapse due to negative pressure, respiratory movement, heartbeat, and cough.

CT of intrathoracic CEH generally shows a lesion with heterogeneous content, a thick wall, and calcifications, suggestive of past inflammation [13,14]. However, differentiation from malignant tumors, such as empyema-associated lymphoma, soft tissue sarcoma, and squamous cell carcinoma, is difficult using CT alone [5].

MRI is an effective diagnostic modality for CEH. On MRI, CEH has been reported to have a low signal-intensity peripheral capsule and central contents with signal intensities ranging from high to low, the ‘mosaic sign [15].’ Varying signal intensities indicate the presence of fresh and old blood caused by repeated bleeding over time [15]. A pseudocapsule with low signal intensity on T1- and T2-weighted images is also a characteristic of CEH [16]. The patient's medical history is important because most patients with CEH have a history of trauma or surgery. Percutaneous needle biopsy should be avoided because massive bleeding and hemorrhagic shock can occur following needle aspiration [17]. In the present case, the patient's clinical course and MRI findings were essential in diagnosing CEH.

To date, FDG-PET imaging features of CEH have not been clearly established. In the present case, FDG uptake was only observed in the peripheral rim of the space-occupying mass, most of which showed no uptake. The peripheral portion of a CEH arising from the thorax or pelvic cavity tends to take up FDG within a SUVmax range of 3.1 to 5.5 [[18], [19], [20]]. Although the same pattern may be observed in malignant tumors with central necrosis, the uptake of FDG in the peripheral rim of the mass should be recognized as a characteristic of CEH.

When intrathoracic CEH is left untreated, the gradually expanding hematoma may compress adjacent organs, resulting in symptoms [21] and sudden death [9]. Therefore, early surgical resection is recommended as the treatment of choice to prevent complications. Complete resection of the hematoma, including the capsule, is desirable [22] because incomplete resection might result in recurrence [10]. However, in some cases the capsule adheres strongly to the surrounding tissue. In the present case, the capsule strongly adhered to the descending aorta, pericardium, left diaphragm, and left lung parenchyma, and we were unable to completely remove the capsule surrounding the descending aorta. Although the hematoma has not recurred, careful long-term follow-up is needed.

Recently, some clinicians have reported the usefulness of preoperative embolization of the arterial supply to the CEH because neovascularization beneath the capsule and rigid adhesion to surrounding tissues often cause massive bleeding in the perioperative and postoperative periods [10,22,23]. Although preoperative embolization was performed in the present case, a significant amount of bleeding was observed when dissecting the adhesions. Therefore, despite preoperative embolization, surgery should only be performed after appropriate presurgical preparations for autologous blood donation have been undertaken. Kuwata et al. recommended a palliative removal in combination with preoperative arterial embolization for a patient who were unable to tolerate complete removal [24]. Nakae et al. also reported that preoperative embolization of the supplying vessels was useful in preventing CEH recurrence [25]. However, longer follow-up and additional cases are needed if this strategy will represent an alternative treatment for CEH.

## 4 Conclusion

In this report, we described the radiological features of intrathoracic CEH in detail. CEH is difficult to distinguish from malignant tumors using CT alone. MRI is a good diagnostic modality for CEH and shows a mixture of low- and high-intensity areas on T2-weighted images. In addition, the patient's medical history is important because most cases of CEH have a history of trauma or surgery. To diagnose intrathoracic CEH, it is essential to consider the patient's clinical course and MRI findings.

## Abbreviations

CEH: chronic expanding hematoma
CT: Computed tomography
MRI: magnetic resonance imaging
PET: Positron emission tomography
FDG: 2-[18 F] fluoro-2-deoxy-d-glucose
SUV: standardized uptake value

## Consent

Written informed consent was obtained from the patient for publication of this case report and accompanying images. A copy of the written consent is available for review by the Editor-in-Chief of this journal on request.

## Ethics approval

As it is a case report, ethical approval is exempted by University of Miyazaki Hospital.

## Funding

The authors have no competing interests to declare.

## Author contribution

Dr. Mayu Inomata is the main author and she has designed this report.

Dr. Shoei Kuroki, Dr. Hiroshi Nakada, and Dr. Fumiya Kawano have reviewed.

Dr. Ryo Maeda is the writer of this article and corresponding author.

## Guarantor

Dr. Ryo Maeda accepts all responsibility of this article.

## Research registration number

Not applicable.

## Conflict of interest statement

All authors have read and approved the final manuscript.
